# Waste Couture: Environmental Impact of the Clothing Industry

**DOI:** 10.1289/ehp.115-a449

**Published:** 2007-09

**Authors:** Luz Claudio

On a Saturday afternoon, a group of teenage girls leaf through glossy fashion magazines at a New Jersey outlet mall. Shopping bags brimming with new purchases lay at their feet as they talk excitedly about what’s in style to wear this summer. Far away in Tanzania, a young man proudly wears a T-shirt imprinted with the logo of an American basketball team while shopping at the local *mitumba* market for pants that will fit his slender figure. Although seemingly disparate, these two scenes are connected through the surprising life cycle of clothing.

How does a T-shirt originally sold in a U.S. shopping mall to promote an American sports team end up being worn by an African teen? Globalization, consumerism, and recycling all converge to connect these scenes. Globalization has made it possible to produce clothing at increasingly lower prices, prices so low that many consumers consider this clothing to be disposable. Some call it “fast fashion,” the clothing equivalent of fast food.

Fast fashion provides the marketplace with affordable apparel aimed mostly at young women. Fueling the demand are fashion magazines that help create the desire for new “must-haves” for each season. “Girls especially are insatiable when it comes to fashion. They have to have the latest thing, always. And since it is cheap, you buy more of it. Our closets are full,” says Mayra Diaz, mother of a 10-year-old girl and a buyer in the fashion district of New York City. Disposable couture appears in shopping mall after shopping mall in America and Europe at prices that make the purchase tempting and the disposal painless.

Yet fast fashion leaves a pollution footprint, with each step of the clothing life cycle generating potential environmental and occupational hazards. For example, polyester, the most widely used manufactured fiber, is made from petroleum. With the rise in production in the fashion industry, demand for man-made fibers, especially polyester, has nearly doubled in the last 15 years, according to figures from the Technical Textile Markets. The manufacture of polyester and other synthetic fabrics is an energy-intensive process requiring large amounts of crude oil and releasing emissions including volatile organic compounds, particulate matter, and acid gases such as hydrogen chloride, all of which can cause or aggravate respiratory disease. Volatile monomers, solvents, and other by-products of polyester production are emitted in the wastewater from polyester manufacturing plants. The EPA, under the Resource Conservation and Recovery Act, considers many textile manufacturing facilities to be hazardous waste generators.

Issues of environmental health and safety do not apply only to the production of man-made fabrics. Cotton, one of the most popular and versatile fibers used in clothing manufacture, also has a significant environmental footprint. This crop accounts for a quarter of all the pesticides used in the United States, the largest exporter of cotton in the world, according to the USDA. The U.S. cotton crop benefits from subsidies that keep prices low and production high. The high production of cotton at subsidized low prices is one of the first spokes in the wheel that drives the globalization of fashion.

## Bringing Clothes to Market Fast, the Global Way

Much of the cotton produced in the United States is exported to China and other countries with low labor costs, where the material is milled, woven into fabrics, cut, and assembled according to the fashion industry’s specifications. China has emerged as the largest exporter of fast fashion, accounting for 30% of world apparel exports, according to the UN Commodity Trade Statistics database. In her 2005 book *The Travels of a T-Shirt in the Global Economy*, Pietra Rivoli, a professor of international business at the McDonough School of Business of Georgetown University, writes that each year Americans purchase approximately 1 billion garments made in China, the equivalent of four pieces of clothing for every U.S. citizen.

According to figures from the U.S. National Labor Committee, some Chinese workers make as little as 12–18 cents per hour working in poor conditions. And with the fierce global competition that demands ever lower production costs, many emerging economies are aiming to get their share of the world’s apparel markets, even if it means lower wages and poor conditions for workers. Increasingly, clothing being imported to the United States comes from countries as diverse as Honduras and Bangladesh.

Once bought, an estimated 21% of annual clothing purchases stay in the home, increasing the stocks of clothing and other textiles held by consumers, according to *Recycling of Low Grade Clothing Waste*, a September 2006 report by consultant Oakdene Hollins. The report calls this stockpiling an increase in the “national wardrobe,” which is considered to represent a potentially large quantity of latent waste that will eventually enter the solid waste stream. According to the EPA Office of Solid Waste, Americans throw away more than 68 pounds of clothing and textiles per person per year, and clothing and other textiles represent about 4% of the municipal solid waste. But this figure is rapidly growing.

## Everything Old Is New Again

In her book *Waste and Want: A Social History of Trash*, Susan Strasser, a professor of history at the University of Delaware, traces the “progressive obsolescence” of clothing and other consumer goods to the 1920s. Before then, and especially during World War I, most clothing was repaired, mended, or tailored to fit other family members, or recycled within the home as rags or quilts. During the war, clothing manufacturers reduced the varieties, sizes, and colors of their productions and even urged designers to create styles that would use less fabric and avoid needless decoration. The government’s conservation campaign used slogans such as “Make economy fashionable lest it become obligatory” and resulted in an approximate 10% reduction in the production of trash.

However, the spirit of conservation did not last long; by the mid-1920s consumerism was back in style. Industrialization grew in the twentieth century, providing the means of increased production of all consumer goods. During World War II, consumption rose with increased employment as the United States mobilized for the war. The production and consumption of many household goods, including clothing, grew by 10–15% even in the middle of the war and continues to expand to this day.

Industrialization brought consumerism with it as an integral part of the economy. Economic growth came to depend on continued marketing of new products and disposal of old ones that are thrown away simply because stylistic norms promote their obsolescence. When it comes to clothing, the rate of purchase and disposal has dramatically increased, so the path that a T-shirt travels from the sales floor to the landfill has become shorter.

Yet even today, the journey of a piece of clothing does not always end at the landfill. A portion of clothing purchases are recycled mainly in three ways: clothing may be resold by the primary consumer to other consumers at a lower price, it may be exported in bulk for sale in developing countries, or it may be chemically or mechanically recycled into raw material for the manufacture of other apparel and non-apparel products.

Domestic resale has boomed in the era of the Internet. Many people sell directly to other individuals through auction websites such as eBay. Another increasingly popular outlet is consignment and thrift shops, where sales are growing at a pace of 5% per year, according to the National Association of Resale and Thrift Shops.

The U.S. government offers tax incentives for citizens who donate household goods to charities such as the Salvation Army and Goodwill Industries, which salvage a portion of clothing and textiles that would otherwise go to landfills or incinerators. The trend of increased purchasing of clothing and other household goods has served the salvage charities well. For instance, since 2001 Goodwill Industries has seen a 67% increase in its sale of donated goods, most of it clothing. Figures from the National Association of Resale and Thrift Shops put Goodwill’s sales of donated goods at thrift shops at more than $1.8 billion in 2006.

A 2006 survey conducted by America’s Research Group, a consumer trends research firm, found that about 12–15% of Americans shop at consignment or resale stores. The Council for Textile Recycling estimates that 2.5 billion pounds of postconsumer textile waste (which includes anything made of fabric) is thus collected and prevented from entering directly into the waste stream. This represents 10 pounds for every person in the United States, but it is still only about 15% of the clothing that is discarded.

## Handling the Overflow

Only about one-fifth of the clothing donated to charities is directly used or sold in their thrift shops. Says Rivoli, “There are nowhere near enough people in America to absorb the mountains of castoffs, even if they were given away.”

So charities find another way to fund their programs using the clothing and other textiles that can’t be sold at their thrift shops: they sell it to textile recyclers at 5–7 cents per pound. Since 1942, the Stubin family of Brooklyn, New York, has owned and operated Trans-America Trading Company, where they process more than 12 million pounds of postconsumer textiles per year. Trans-America is one of the biggest of about 3,000 textile recyclers in the United States. At its 80,000-square-foot sorting facility, workers separate used clothing into 300 different categories by type of item, size, and fiber content. According to figures from Trans-America, about 30% of these textiles are turned into absorbent wiping rags for industrial uses, and another 25–30% are recycled into fiber for use as stuffing for upholstery, insulation, and the manufacture of paper products.

About 45% of these textiles continue their life as clothing, just not domestically. Certain brands and rare collectible items are imported by Japan, the largest buyer in terms of dollars of vintage or American high-end fashion. Clothing that is not considered vintage or high-end is baled for export to developing nations. Data from the International Trade Commission indicate that between 1989 and 2003, American exports of used clothing more than tripled, to nearly 7 billion pounds per year. Used clothing is sold in more than 100 countries. For Tanzania, where used clothing is sold at the *mitumba* markets that dot the country, these items are the number one import from the United States.

Imported apparel from America and Europe is bought in 100-pound bales of mixed clothing by small entrepreneurs. Like opening a piñata, these merchants sort through the contents of the bales to see whether their investment has paid off. Prices are set according to the latest fashions, the condition of the clothing, and its desirability. For example, men’s light slacks in perfect condition and in waist sizes in the low 30s fetch a premium price of $5.00. T-shirts sell well, especially those with logos from winning sports teams or recognizable athletic gear companies.

Because women in the West tend to buy much more clothing and discard it more often than men, the world supply of used women’s clothing is at least seven times that of men’s. Thus, in the *mitumba* markets around Tanzania, men’s clothing generally costs four to five times more than similar women’s clothing. Winter clothes, although generally more expensive to produce, command the least value in the secondhand African markets. Companies such as Trans-America are therefore seeking to expand into colder climes such as Eastern Europe.

Observers such as Rivoli predict that the trend toward increasing exports of used clothing to developing countries will continue to accelerate because of the rise of consumerism in the United States and Europe and the falling prices of new clothing. There are detractors to this view, however. For example, the Institute for Manufacturing at Cambridge University issued a report in 2006 titled *Well Dressed? The Present and Future Sustainability of Clothing and Textiles in the United Kingdom*, in which it raised concerns that trade in secondhand clothes in African countries inhibits development of local industries even as it creates employment in these countries. And the authors of *Recycling of Low Grade Clothing Waste* warn that in the long run, as prices and quality of new clothing continue to decline, so too will the demand for used clothing diminish. This is because in the world of fast fashion, new clothing could be bought almost as inexpensively as used clothing. Even so, says Rivoli, “Continued rampant consumerism as well as changing waste disposal practices would seem to ensure a growing supply of American used clothing for the global market.”

## Fashion Forward

To address the environmental impacts of fast fashion at its source, and to find a niche in this increasingly competitive market, some manufacturers are aiming to develop “eco-fashions.” The International Standards Organization (ISO) has defined eco-fashions as “identifying the general environmental performance of a product within a product group based on its whole life-cycle in order to contribute to improvements in key environmental measures and to support sustainable consumption patterns.” The ISO is developing standards for a labeling system to identify garments that meet criteria as environmentally friendly. However, even without such specific standards for what constitutes an environmentally friendly garment, industry is taking a broadening diversity of approaches.

One approach has been to use sustainably grown cotton, hemp, bamboo, and other fiber crops that require less pesticides, irrigation, and other inputs. Organic cotton is grown in at least 12 countries. Figures provided by the Organic Trade Association 2004 Manufacturer Survey show that the sale of organic cotton fiber grew by an estimated 22.7% over the previous year. Sales of organic cotton women’s clothing grew by a healthy 33%. However, organic cotton represents only 0.03% of worldwide cotton production. This figure may grow as retailers begin to expand their selections of organic cotton apparel. In 2004, Wal-Mart, America’s largest retailer, began selling organic cotton women’s shirts at its Sam’s Club stores. Today the company is the world’s largest buyer of organic cotton, offering several lines of organic cotton apparel and bedding goods in its Wal-Mart and Sam’s Club stores. By the time of a 31 July 2006 report on CNNMoney.com, the company had sold 5 million units of organic cotton ladies’ apparel.

According to *Well Dressed?*, about 60% of the energy used in the life cycle of a cotton T-shirt is related to postpurchase washing and drying at high temperatures; transportation constitutes only a small portion of the energy profile to produce a cotton product. As for whether it is better to buy locally produced garments, the report argues that this approach would cut severely into the livelihood of peoples in developing countries where the products are now being manufactured.

More innovative eco-fashions are being developed and made available to consumers at different levels of the fashion spectrum, from casual clothing to haute couture. Patagonia, a major retailer in casual wear, has been selling fleece clothing made from postconsumer plastic soda bottles since 1993. This recycling process takes clear plastic bottles made of polyethylene terephthalate (PET), melts them, and reconfigures them into fibers that can be woven into fabrics and other applications. Patagonia is one of the first and largest clothing retailers to use this material. The company estimates that between 1993 and 2006 it saved 86 million soda bottles from ending up in the landfill. Patagonia also recycles its cotton T-shirts through Italian company Calamai Functional Fabrics. According to Trailspace.com, an outdoor gear information site, recycling cotton saves 20,000 liters of water per kilogram of cotton, a water-intensive crop.

Another approach is the use of polymers created from plant-based materials. One such material trademarked by Cargill, Ingeo, is made of corn by-products that are fermented and transformed into polylactide. This polymer is spun into fibers and woven into fabrics that, under strictly managed circumstances, could be composted (polylactide, marketed under the name NatureWorks PLA, is also fashioned into wraps, rigid food and beverage containers, coated papers and boards, and other packaging applications). Versace is one of the haute couture designer clothing firms that have used Ingeo in their collections.

Other retailers large and small are taking different steps to appeal to the environmentally conscious consumer. Tesco, the largest British retailer, has commissioned a study by Oxford University toward developing a Sustainable Consumption Institute to establish a system to label every product sold by Tesco on the basis of its carbon emission footprint. This plan was highlighted at the 2007 Association of Suppliers to the British Clothing Industry Conference. Many in the industry think such efforts are not only good for the environment, but also makes good business sense. Hana Ben-Shabat, vice president of goods and retail practice at AT Kearney, a management consulting firm that works with fashion industry suppliers, stated in a presentation at the conference that “being green and ethical is no longer an option, it is [an economic] necessity.”

In the European Union, the Registration, Evaluation, Authorisation and Restriction of Chemicals (REACH) regulations enacted 1 June 2007 require clothing manufacturers and importers to identify and quantify the chemicals used in their products. These regulations may even require manufacturers to inform consumers about potentially hazardous chemicals that may be present in their products and can leach out, such as often happens with dyes (details of how the regulations will be implemented are still being worked out). Actual end products are governed by stipulations of the European Equipment and Product Safety Act, which regulates the use of heavy metals, carcinogenic dyes, and other toxics used in textile manufacture. Additional consumer protection is offered by the European Union’s Öko-Tex Standard 100, a testing and certification program established in 1992. The standard gives the textile and clothing industry uniform guidance for the potential harm of substances in raw materials as well as finished products, and every stage in between—these include regulated substances as well as substances that are believed to be harmful to health but are not yet regulated (such as pesticides). The standard also governs elements such as colorfastness and pH value.

Such regulations and standards, coupled with increasing consumer awareness about less toxic and sustainable products, may provide some impetus to revolutionize the garment industry. However, the biggest impacts for increasing sustainability in the clothing industry rests with the consumer. Using detergents that work well at lower temperatures, extending the usable life of garments, purchasing fewer and more durable garments, and recycling these garments into the used clothing market or into other garment and nongarment products all would contribute to increasing sustainability. Consumer awareness about the fate of clothing through its life cycle may be the best hope for sustainability in the fashion industry.

## Figures and Tables

**Figure:**
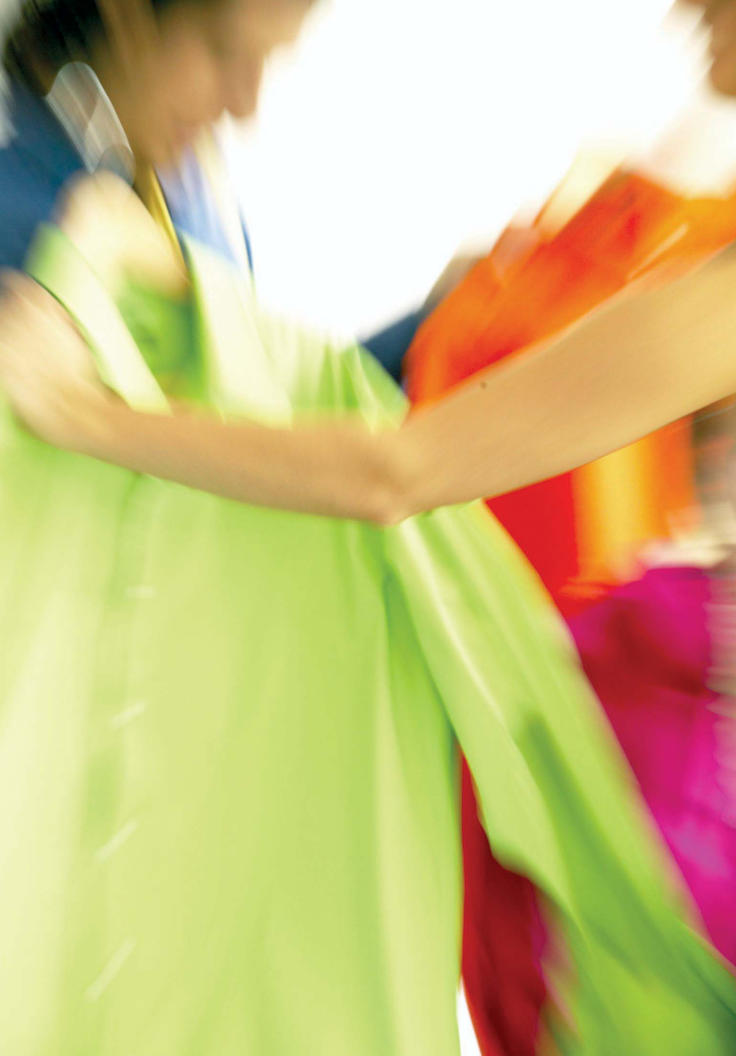


**Figure:**
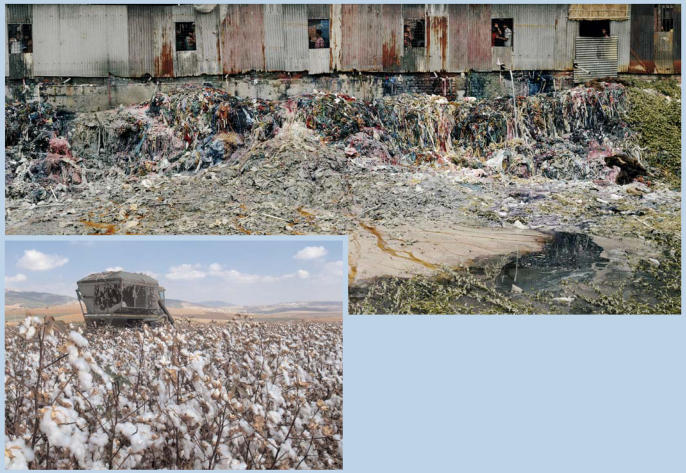
Each step of the clothing production process carries the potential for an environmental impact. For example, conventionally grown cotton, one of the most popular clothing fibers, is also one of the most water- and pesticide-dependent crops (a view disputed by Cotton Incorporated, a U.S. cotton growers' group). At the factory stage, effluent may contain a number of toxics (above, waste products from a garment factory in Dhaka, Bangladesh, spill into a stagnant pond).

**Figure:**
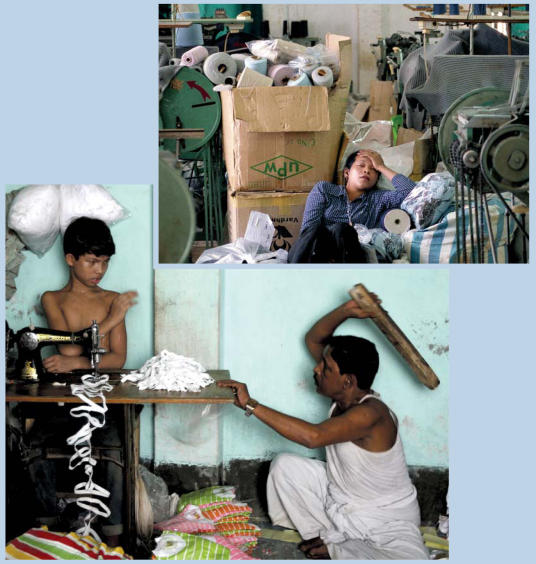
Fierce global competition in the garment industry translates into poor working conditions for many laborers in developing nations. (top) A worker in Phnom Penh, Cambodia, rests on the floor of a garment factory. More than 2,000 young women work in this factory, producing clothes for shops in Europe and North America. (bottom) The owner of a textile factory in Dhaka threatens a child laborer, who works for 10 hours a day to earn US$1.

**Figure:**
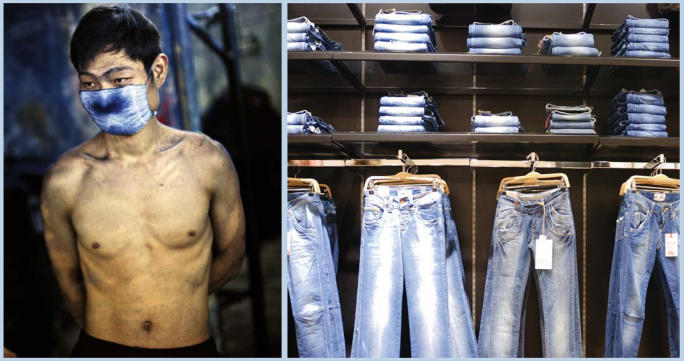
A textile worker takes a break at dawn after sanding jeans all night at a clothing factory in Guangdong Province, China. The blue dust from the jeans is a heavy irritant to the lungs. The factory where this worker is employed uses a wear-and-tear process to achieve the fashionable distressed look for the approximately 10,000 pairs of jeans it produces every day. Thousands of workers labor around the clock scrubbing, spraying, and tearing jeans in order to meet the production demand. China is one of the world's largest producers of jeans.

**Figure:**
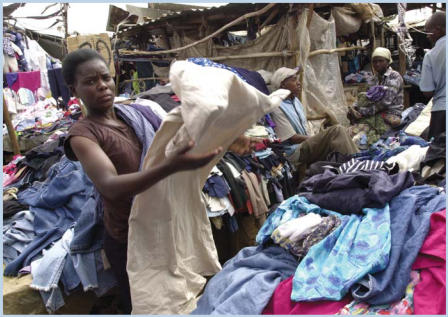
A woman shops at a *mitumba* (Swahili for "secondhand") market in Nairobi, Kenya. Middlemen purchase bales of clothing at a set price to resell at the *mitumba* market. Sometimes the bales contain prize garments, other times less desirable items, and the clothing may be sold by the piece or by weight. People often buy large amounts of clothing to resell yet again in smaller markets outside the city.

**Figure:**
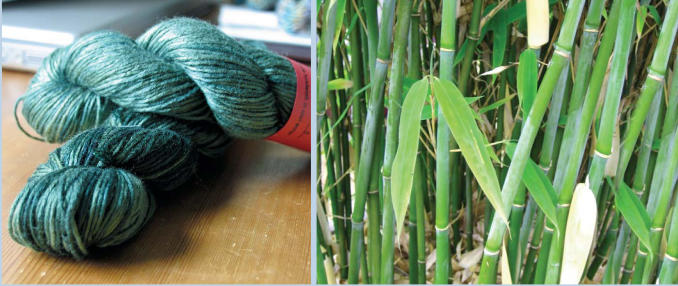
Alternative fibers such as bamboo (in yarn and original form, above) and hemp (of a variety that produces only a tiny amount of the psychoactive component found in cannabis) are coming into greater use in so-called eco-fashions. In February 2005, as part of New York City’s Fashion Week, retailer Barneys New York and the nonprofit Earth Pledge sponsored FutureFashion, a showcase of environmentally friendly apparel.

